# The prevalence of electrocardiographic early repolarization in an adult cohort with chronic kidney disease and its impact upon all-cause mortality and progression to dialysis

**DOI:** 10.3389/fphys.2013.00127

**Published:** 2013-05-31

**Authors:** Reza Hajhosseiny, Ronak Rajani, Kaivan Khavandi, Frédéric A. Sebag, Soudeh Mashayekhi, Matthew Wright, David Goldsmith

**Affiliations:** ^1^MRC Centre for Transplantation and Renal Unit, Guy's and St. Thomas' NHS Foundation Trust, King's College Academic Health PartnersLondon, UK; ^2^BHF Centre of Cardiovascular Excellence, Guy's and St. Thomas' NHS Foundation Trust, King's College Academic Health PartnersLondon, UK

**Keywords:** early repolarization, chronic kidney disease, sudden cardiac death, dialysis, mortality

## Abstract

**Background:** Electrocardiographic early repolarization (ER) occurring in <5% of general/atherosclerotic populations, is a marker of sudden cardiac death (SCD). The prevalence of ER in chronic kidney disease (CKD) patients, in whom SCD is common, is unknown. We aimed to determine the prevalence, contributing factors, and relationship of ER to all-cause mortality and progression to dialysis in CKD patients.

**Methods:** A retrospective study of 197 patients with stage 3–5 CKD. Full demographic data were collected including cardiovascular risk factors and history. All patients underwent a 12-lead ECG, analysed for the presence of ER and other ECG findings. ER was defined as elevation of the QRS-ST junction (J point) by at least 0.1 mV from baseline with slurring/notching of the QRS complex. The primary and secondary endpoints were all cause mortality and progression to dialysis respectively at 1 year. To control for the effects of CKD, we evaluated the ECGs of 39 healthy renal transplant donors (RTD).

**Results:** CKD patients had a mean age of 61.5 (±16.1). Prevalence of ER in pre-dialysis patients with CKD stage 4 and 5 was higher than in RTD (26.4 vs. 7.7%, *p* = 0.02). ER frequency increased with CKD stage (stage 3: 7.7%, stage 4: 29.7%, and pre-dialysis stage 5: 24.6%), but decreased in dialysis patients (13%). On multivariate analysis only the QRS duration was a significant independent predictor of ER (OR 0.97, 95% CI, 0.94–0.99, *p* = 0.01). At 1-year follow-up, there were 24 (12%) deaths in the patients with CKD of whom 5 (21%) had ER. ER was not a predictor of all cause mortality (*p* = 1.00) and had no effects on the rate of progression to dialysis (*p* = 0.67).

**Conclusions:** ER is more common in pre-dialysis CKD patients, compared to healthy RTD but is not associated with increased 1-year mortality or entry onto dialysis programs. Further longitudinal studies are indicated to determine whether this increased prevalence of ER is associated with the rate of SCD seen in this population.

## Introduction

The renal and cardiovascular systems have a unique and intricate inter-relationship, with disease or dysfunction in one organ frequently leading to injury in the other. This complex interaction has led to the use of the term “cardio-renal syndrome” (Ronco et al., 2009; Hajhosseiny et al., 2013). Overall, for subjects with chronic kidney disease (CKD) stage 3, it is more likely that a patient will develop cardiovascular disease (CVD) than progress to dialysis-requiring renal failure (CKD stage 5D; Bleyer et al., 2006; Hajhosseiny et al., 2013). Sudden cardiac death (SCD) is particularly prevalent amongst patients with CKD, with estimates ranging from 25 to 60% (Herzog, 2003; Pun et al., 2009). In dialysis patients, the incidence of SCD is very high; eclipsing other causes of cardiac death, and rises with both the duration of time that the patient has been on a dialysis program, as well as the severity and frequency of dialysis-associated electrolyte imbalances (Karnik et al., 2001; Bleyer et al., 2006).

Until recently, electrocardiographic early repolarization (ER) was considered a benign finding on a patient's electrocardiogram (ECG). However, a number of recent studies have suggested that ER may represent an independent marker of sudden arrhythmic cardiac arrest in otherwise healthy individuals (Haissaguerre et al., 2008; Ghosh et al., 2010; Sinner et al., 2010; Tikkanen et al., 2012). Despite this, it is currently unknown whether or not there is a similar increased prevalence of ER in CKD patients in whom SCD is common. The aim of the current study was therefore to determine the prevalence of ER, possible contributory factors, and whether or not ER is related to all-cause mortality and progression to dialysis in patients with CKD.

## Methods

### Patients

We retrospectively studied adults with stage 3–5 CKD referred to the Nephrology department at Guy's and St. Thomas' Hospital between March 2007 and December 2011. Our study population consisted of newly diagnosed patients with CKD, or patients with follow-up appointments. In addition, we also studied 39 adults without CKD to control for the effects of renal failure on ER. These controls were healthy renal transplant donors (RTD) with no documentation of structural heart disease or a history of syncope. Full demographic data pertaining to cardiovascular risk factors, prior cardiac history, current medications, and concurrent co-morbidities were recorded along with recent laboratory results (renal profile, serum calcium, and hs C-reactive protein levels). Cardiovascular risk factors were determined by pre-set defined criteria. Hypertension was defined as a systolic blood pressure of >140 mmHg, a diastolic blood pressure of >90 mmHg, or antihypertensive drug use. Smoking was defined as a current smoker or past heavy smoker (>20 package-years). Diabetes mellitus was defined as a previously established diagnosis, insulin, or oral hypoglycemic therapy, fasting glucose of >126 mg/dL, or non-fasting glucose of >200 mg/dL. Family history of coronary artery disease was defined as myocardial infarction, coronary revascularization, or SCD in a first-degree relative <65 years old. A 12-lead ECG was performed on all patients and the primary inclusion criteria were based on CKD severity and the availability of a recent adequate ECG recording in their clinical file. Patients with permanent pacemakers were excluded from the study. All patients were followed up for a minimum time period of 12-months from the data of the first ECG. Written informed consent was obtained from the 19 haemodialysis patients for obtaining an ECG before and after dialysis and the study was granted institutional ethics committee approval in accordance with the Helsinki Declaration (1964, amended in 1975 and in 1984).

### ECG acquisition and analysis

**Figure 1:**
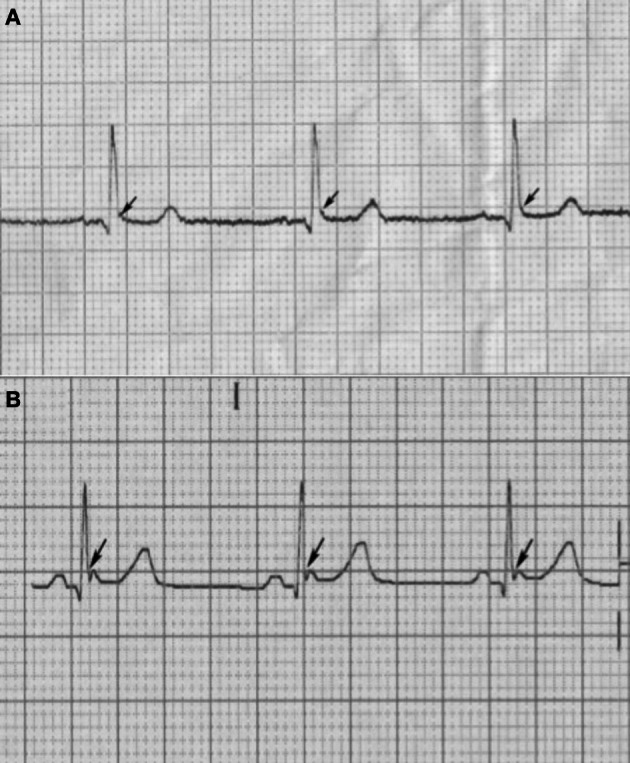
**The identification of ER from the 12-lead electrocardiogram. (A)** Shows a typical example of a slurred patterned ER (arrows) while **(B)** shows the appearance of a notched ER (arrows).

All patients underwent a 12-lead ECG. These were fully interrogated by two electrophysiologists blinded to the clinical data for relevant electrical intervals (PR, QRS, and QTc durations), and for bundle branch block, the cardiac axis, left ventricular hypertrophy (LVH), and ER. The presence or absence of LVH was assessed according to the Sokolow–Lyon criteria and the QT interval was corrected for heart rate according to Bazett's formula. Early repolarization was defined as an elevation of the QRS-ST junction (J point) in at least two leads. The amplitude of J-point elevation had to be at least 1 mm (0.1 mV) above the baseline level either as QRS slurring (a smooth transition from the QRS segment to the ST segment—Figure 1A) or notching (a positive J deflection inscribed on the S wave—Figure 1B) in the inferior leads (II, III, and aVF), lateral leads (I, aVL, and V4–V6), or both. The anterior precordial leads (V1–V3) were excluded from the analysis to avoid the inclusion of patients with right ventricular dysplasia or Brugada syndrome. In order to account for the effects of dialysis on ER, we obtained an ECG recording from 19 patients on hemodialysis immediately prior to the start of their dialysis session, and followed this up with the acquisition of another ECG immediately after their dialysis session had finished.

### Follow up

In order to investigate the prognostic and predictive value of ER, all patients (CKD and controls) were followed up for 12 months from the start of the study. The primary endpoint was all-cause mortality (all cause) and the secondary endpoint, the new commencement of dialysis.

### Statistical analysis

All continuous variables included in the analysis are presented as mean ± SD. Variables with non-normal distributions are presented as median with range. Univariate analyses were performed on continuous variables using the two-sample *t*-test for normally distributed variables and the Mann–Whitney U test for non-normally distributed data. Spearman's correlation coefficient was used to assess the relationship between continuous variables. Multivariable logistic regression was used to determine the predictors of ER using age, presence of COPD, mineralocorticoid receptor antagonist use, heart rate, PR interval, QRS interval, and dialysis as covariates. Statistical significance for all analyses was set at the 5% level. All data were collected and analyzed using SPSS for MAC (Version 19, IBM, Somers, NY, USA).

## Results

The baseline demographics of the population are given in Table 1. There were 202 patients with CKD stage 3–5 who were approached for inclusion into the study. Of these, 5 patients were excluded due to the presence of a permanent cardiac pacemaker. In the remaining cohort of 197 patients, the ethnic origin was Caucasian in 121 (61%), Afro-Caribbean in 60 (31%), and South Asian in 16 (8%). There was a prior cardiac history present in 62 (32%) patients [8 (4%) congestive cardiac failure, 47 (24%) coronary artery disease, 14 (16%) myocardial infarction, 8 (4%) valvular heart disease and 14 (7%) atrial fibrillation].

**Table 1:** **Baseline characteristics of patients with CKD**.

	**CKD (*N* = 197)**
Age (years)	61.5 ± 16.1
Gender (male) *n* (%)	113 (57)
eGFR (mL/min/1.73 m^2^)	14.0 (10.0–17.3)
**CKD STAGE *n* (%)**	
3	13 (6.6)
4	37 (18.8)
5 no dialysis	69 (35.0)
5 dialysis	78 (39.6)
Renal Transplant *n* (%)	16 (8.1)
Duration of CKD (months)	25 (12–54)
Stroke *n* (%)	19 (9.6)
COPD *n* (%)	11 (5.6)
**CARDIOVASCULAR RISK FACTOR *n* (%)**	
Diabetes	77 (39.1)
BMI (kg/m^2^)	28.8 ± 6.6
Current smoker	4 (2)
Dyslipidaemia	33 (16.8)
Hypertension	133 (67.5)
Systolic blood pressure (mmHg)	145 ± 25
Diastolic blood pressure (mmHg)	75 ± 15
**MEDICATIONS *n* (%)**	
β blockers	73 (37.1)
Diuretics	72 (36.5)
Aldosterone antagonist	4 (2.0)
ACE inhibitors	53 (26.9)
ARB	49 (24.9)
CCB	4 (2.0)
Amiodarone	1 (0.5)
Statins	106 (53.8)
Erythropoietin	112 (56.9)
**BIOCHEMICAL**	
Potassium (mmol/L)	4.7 ± 0.7
Calcium (mmol/L)	2.3 ± 0.3
Phosphate (mmol/L)	1.4 ± 0.4
Parathormone (PTH)	191 (97–396)
CRP	6 (5–20)
Hemoglobin (g/dL)	10.8 ± 1.9
**ELECTROCARDIOGRAM**	
Heart rate (bpm)	76 ± 17
PR duration (ms)	162 ± 49
QRS duration (ms)	92 ± 20
QRS axis (°)	17.3 ± 41.5
Right BBB *n* (%)	11 (5.6)
Left BBB *n* (%)	6 (3.0)
Electrical LVH *n* (%)	33 (16.8)
QTc duration (ms)	424 ± 32

Abbreviations *ACE-I* *angiotensin converting enzyme inhibitors* *ARB* *angiotensin receptor blockers* *BMI* *body mass index* *CKD* *chronic kidney disease* *COPD* *chronic obstructive pulmonary disease* *CRP* *C-reactive protein* *PTH* *parathyroid hormone*.

### Prevalence of early repolarization in patients with and without CKD

Table 2 gives the comparison of baseline demographics between the 197 patients with CKD and the 39 healthy renal donors who represented the control group. The healthy renal donors were of a younger age and were predominantly Caucasian in ethnic origin. Although there was a tendency to an increased prevalence of ER in the CKD population (19.8%) compared with the renal donors (7.7%), this did not reach statistical significance (*P* = 0.07). There was no difference observed in the magnitude or distribution of J-point elevation. Prevalence of ER in pre-dialysis patients with CKD stage 4 and 5 was higher than in the RTD (26.4 vs. 7.7%, *p* = 0.02). Early repolarization frequency increased with CKD stage (stage 3: 7.7%, stage 4: 29.7%, and pre-dialysis stage 5: 24.6%), but decreased in dialysis patients (13%).

**Table 2:** **Comparison of baseline characteristics of patients with CKD vs. the renal transplant donors**.

	**CKD**	**Donors**	***p***
	**(*n* = 197)**	**(*n* = 39)**	
Age (years)	61.5 ± 16.1	44.0 ± 11.6	<0.0001
**ETHNICITY**			
Caucasian *n* (%)	121 (61.4)	35 (89.7)	0.0001
Afro-Caribbean *n* (%)	60 (30.5)	1 (2.6)	
Asian *n* (%)	16 (8.1)	3 (7.7)	
Gender (male) *n* (%)	113 (57.4)	20 (51.3)	0.48
Height (cm)	167 ± 10	170 ± 9	0.04
Weight (kg)	81 ± 21	76 ± 13	0.13
eGFR (mL/min/1.73 m^2^)	14 (10–20)	85 (79–92)	<0.0001
Early repolarization *n* (%)	39 (19.8)	3 (7.7)	0.07
**PATTERN *n* (%)**			
Slurred	26 (66.7)	2 (66.7)	1.00
Notch	13 (33.3)	1 (33.3)	
**J POINT ELEVATION *n* (%)**			
≥0.1 and <0.2 mV	38 (97.4)	3 (100.0)	1.00
≥0.2 mV	1 (2.6)	0 (0.0)	
**LOCALIZATION *n* (%)**			
Inferior	16 (41.0)	0 (0.0)	0.22
Lateral	14 (35.9)	3 (100.0)	
Inferior and lateral	9 (23.1)	0 (0.0)	

Abbreviations *eGFR* *estimated glomerular filtration rate*.

### Predictors of early repolarization

In the CKD group of patients, age, gender, ethnicity, prior cardiac history, cardiovascular risk factors, eGFR, and CKD stage were unrelated to the presence of ER (Table 3). Similarly, there was no relationship to the use of cardiac medications or serum electrolytes and biochemistry. There were however a number of ECG predictors of ER. Patients with ER had lower heart rates (HR with ER 71 ± 15 BPM vs. 77 ± 17 without ER, *p* = 0.03), shorter QRS durations (QRS duration with ER 84 ± 11 vs. 94 ± 21 without ER, *p* < 0.01), and shorter QTc durations (QTc duration with ER 412 ± 22 vs. 427 ± 34 without ER, *p* < 0.001). On multivariate analysis (Table 4) with ER as the dependent variable and age, presence of COPD, mineralocorticoid receptor antagonist use, heart rate, PR interval, QRS interval, and dialysis as covariates, only the QRS duration remained a significant independent predictor of ER (OR 0.97, 95% CI, 0.94–0.99, *p* = 0.01).

**Table 3:** **Comparison of baseline characteristics of adults with ER vs. those without ER**.

	**Overall**	**ER**	**No ER**	***p***
	**(*N* = 197)**	**(*N* = 39)**	**(*N* = 158)**	
Age (years)	61.5 ± 16.1	58.1 ± 17.1	62.3 ± 15.8	0.14
Male *n* (%)	113 (57.4)	22 (56.4)	91 (57.6)	0.89
**ETHNICITY *n* (%)**				
Caucasian *n* (%)	121(61.4)	74 (61.2)	47 (38.8)	0.91
Afro-Caribbean *n* (%)	60 (30.5)	31 (51.7)	29 (48.3)	
Asian *n* (%)	16 (8.1)	8 (50.0)	8 (50.0)	
**CARDIAC HISTORY *n* (%)**				
Congestive heart failure	8 (4.1)	2 (5.1)	6 (3.8)	0.66
Palpitations	1 (0.5)	1 (2.6)	0 (0)	0.20
Syncope	1 (0.5)	0 (0)	1 (0.6)	1.00
SCD	0 (0)	0 (0)	0 (0)	N/A
Coronary artery disease	47 (23.9)	8 (20.5)	39 (24.7)	0.58
Myocardial infarction	14 (16.1)	1 (2.6)	13 (8.2)	0.31
PCI	18 (9.1)	3 (7.7)	15 (9.5)	0.72
CABG	8 (4.1)	1 (2.6)	7 (4.4)	0.59
Other^*^	26 (13.2)	5 (12.8)	21 (13.3)	1.00
eGFR (mL/min/1.73m^2^)	10.0 ± 7.2	12.0 ± 9.2	10 ± 7–17.0	0.38
**CKD STAGE *n* (%)**				
3	13 (6.6)	1 (2.6)	12 (7.6)	0.07
4	37 (18.8)	11 (28.2)	26 (16.5)	
5 no dialysis	69 (35.0)	17 (43.6)	52 (32.9)	
5 dialysis	78 (39.6)	10 (5.1)	68 (43.0)	
Renal dialysis *n* (%)	78 (39.6)	10 (25.6)	68 (43.0)	0.046
Renal transplant *n* (%)	16 (8.1)	3 (7.7)	13 (8.2)	0.91
CKD duration (months)	25 (12–54)	30 (11–57)	25 (12–64)	0.61
Stroke *n* (%)	19 (9.6)	3 (7.7)	16 (10.1)	0.77
COPD *n* (%)	11 (5.6)	0 (0)	11 (7.0)	0.13
**CV RISK FACTORS *n* (%)**				
Diabetes	77 (39.1)	16 (41.6)	61 (38.6)	0.78
BMI (kg/m^2^)	29 ± 7	29 ± 7	29 ± 7	0.86
Current smoker	4 (2)	1 (2.6)	3 (1.9)	1.00
Dyslipidaemia	33 (16.8)	7 (17.9)	26 (16.5)	0.82
Hypertension	133 (67.5)	25 (64.1)	108 (68.4)	0.61
SBP (mmHg)	145 ± 25	141 ± 22	146 ± 26	0.35
DBP (mmHg)	75 ± 15	76 ± 13	75 ± 15	0.76
**MEDICATIONS *n* (%)**				
β blockers	73 (37.1)	16 (41.0)	57 (36.1)	0.56
Diuretics	72 (36.5)	17 (43.6)	55 (34.8)	0.31
MRA	4 (2.0)	2 (5.1)	2 (1.3)	0.17
ACE inhibitors	53 (26.9)	8 (20.5)	45 (28.5)	0.31
ARB	49 (24.9)	10 (25.6)	39 (24.7)	0.90
CCB	4 (2.0)	0 (0)	4 (2.5)	0.59
Amiodarone	1 (0.5)	0 (0)	1 (0.6)	1.00
Statins	106 (53.8)	19 (48.7)	87 (55.1)	0.48
Erythropoietin	112 (56.9)	18 (46.2)	94 (59.5)	0.13
**BIOCHEMISTRY**				
Potassium (mmol/L)	4.7 ± 0.7	4.8 ± 0.6	4.7 ± 0.8	0.34
Calcium (mmol/L)	2.3 ± 0.3	2.3 ± 0.2	2.3 ± 0.3	0.51
Phosphate (mmol/L)	1.4 ± 0.4	1.4 ± 0.4	1.4 ± 0.4	0.68
Parathormone (PTH)	191 (97–396)	221 (100–368)	183 (97–404)	0.92
CRP	6 (5–20)	6 (5–12)	6 (5–24)	0.30
Hemoglobin (g/dL)	10.8 ± 1.9	10.8 ± 1.9	10.7 ± 1.9	0.82
**ELECTROCARDIOGRAM**				
Heart Rate (bpm)	76 ± 17	71 ± 15	77 ± 17	0.03
PR duration (ms)	162 ± 49	172 ± 29	160 ± 52	0.18
QRS duration (ms)	92 ± 20	84 ± 11	94 ± 21	<0.01
QRS axis (°)	17.3 ± 41.5	27.0 ± 25.0	15.0 ± 44.4	0.03
Right BBB *n* (%)	11 (5.6)	1 (2.6)	10 (6.3)	0.70
Left BBB *n* (%)	6 (3.0)	0 (0)	6 (3.8)	0.60
Electrical LVH *n* (%)	33 (16.8)	5 (12.8)	28 (17.7)	0.46
QTc duration (ms)	424 ± 32	412 ± 22	427 ± 34	<0.001

Abbreviations *BBB* *bundle branch block* *CABG* *coronary artery bypass graft* *CCB* *calcium channel blocker* *DBP* *diastolic blood pressure* *ICD* *implantable cardiac defibrillator* *MRA* *mineralocorticoid receptor antagonist* *PCI* *percutaneous coronary intervention* *SBP* *systolic blood pressure* *SCD* *sudden cardiac death*.

* *Atrial fibrillation, ICD, Valvular heart disease, Ventricular tachycardia*.

**Table 4:** **Multivariate logistic regression analyses for the predictors of ER**.

	***OR* (95% CI)**	***p***
Age	0.98 (0.96, 1.01)	0.20
COPD	0.00 (0.00, 0.00)	1.00
Anti-aldosterone	7.95 (0.40, 158.48)	0.18
Heart rate	0.98 (0.95, 1.00)	0.76
PR interval	1.01 (1.00, 1.02)	0.28
QRS duration	0.97 (0.94, 0.99)	0.01
Dialysis	0.55 (0.24, 1.27)	0.16

### Early repolarization as a predictor of all-cause mortality and progression to dialysis

**Figure 2:**
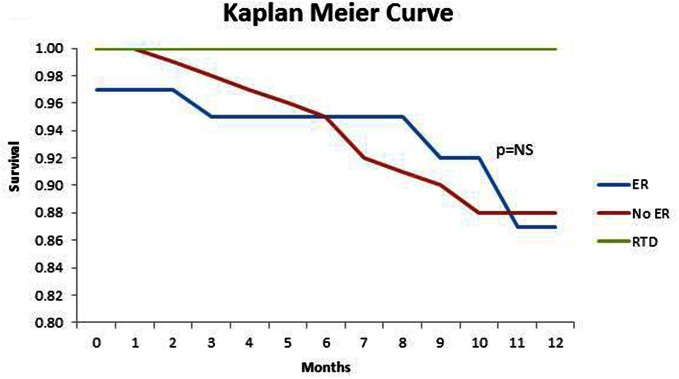
**Kaplan Meier Curve comparing 1-year survival in CKD patients with ER, without ER, and the renal transplant donors (RTD)**.

At 1-year follow-up, there were 24 deaths. There was no difference in mortality between those who had ER 13% (5 of 39 patients) to those who did not 12% (19 of 158 patients), *p* = 1.0 (Figure 2). From the 119 patients who were not already on dialysis at the study commencement, 56 (47%) patients progressed to either peritoneal or hemodialysis. Of the 29 patients with ER who were not yet on dialysis, 15 (52%) progressed to renal replacement therapy, compared with 41 (46%) of the 90 patients without ER (*p* = 0.67).

## Discussion

The main finding of the current study is that there is an increased prevalence of ER in CKD patients (20%) when compared to healthy controls (8%). We also show that the presence of ER is associated to a shorter QRS duration in patients with CKD but that this did not translate to an increased incidence of all-cause mortality or progression to dialysis at 1-year follow-up.

A number of prior studies have suggested a relationship between the presence of ER and SCD. Haissaguerre et al. (2008) conducted a multicentre study of 206 patients resuscitated after an episode of idiopathic ventricular fibrillation. The authors found that there was an increased prevalence of ER (31%) when compared to 412 age, gender, and race matched controls (5%), and that after a median follow-up of 61 months patients with ER had a significantly higher incidence of ventricular fibrillation than those cases without ER (HR 2.1, 95% CI 1.2–3.5, *p* = 0.008). In another study of 432 victims of a SCD from an acute coronary event Tikkanen et al. (2012), showed that there was an increased prevalence of ER (14.4%) when compared to 532 survivors of an acute coronary syndrome (7.9%). Finally, in a study of 1945 individuals aged between 35 and 74 years of age (Sinner et al., 2010), found a greater than 2-fold-increased risk of cardiac mortality in participants with ER compared to individuals without ER.

Although the exact mechanism of ER-induced arrhythmogenicity is still unclear, it has been hypothesized that this may be related to either an increased susceptibility or vulnerability to cardiac arrest in critical ischemic conditions such as acute coronary syndromes (Tikkanen et al., 2012), or to subtle changes in the cardiac action potential (Benito et al., 2010). Early repolarization in its simplest form occurs in Phase 1 of the cardiac action potential and is caused by the cardiac transient outward potassium current (*I*_to_). If a situation arises where there is a reduced density of the *I*_to_ channels in the endocardium compared with epicardium or mid-myocardium (Li et al., 2002), a large *I*_to_ current can occur that results in electrocardiographic ER and large voltage gradients that have the propensity to initiate life threatening arrhythmias (Li et al., 2002; Benito et al., 2010). In addition, an increase in transmural dispersion of ventricular repolarization, which is associated with ER (Karim Talib et al., 2012), has been demonstrated in patients with CKD (Tun et al., 1991; Saravanan and Davidson, 2010). This increase may partially explain the increased prevalence of ER in our CKD population.

In the current study, although we showed an increased prevalence of ER in patients with CKD, this was not associated with either all-cause mortality at 1-year or progression to dialysis. There are a number of potential explanations for this finding. The sample size in the current study was modest in comparison to prior studies and it is possible that any true effect of ER on mortality or progression to dialysis was concealed. It is also possible that CKD patients represent an entirely different cohort of patients to that previously studied and that the presence of ER may not be of prognostic importance. Further studies of larger cohorts of patients with CKD are indicated to clarify the findings of the current study.

Interestingly we also showed on univariate analysis that the presence of ER was related to a slower heart rate and shorter QRS and QTc durations. Although slower heart rates have been shown to be associated with ER (Li et al., 2002; Benito et al., 2010), potentially as a result of time-dependent recovery of *I*_to_ from inactivation (Antzelevitch and Yan, 2010; Benito et al., 2010), the finding of an association of a shorter QRS duration to an increased prevalence of ER is discrepant with prior studies. (Tikkanen et al., 2009, 2012) showed that ER was related to a slight increase in the QRS duration, while Haissaguerre et al. (2008) found no relationship between ER and QRS duration. It is possible that in the current study a broader QRS duration may have masked subtle J point elevation making the significance of this finding difficult to interpret. In keeping with prior reports (Haissaguerre et al., 2008; Watanabe et al., 2010) we also showed that a shorter QTc interval was associated to ER supporting the hypothesis that ER and short QT syndrome may share common cardiac channel genetic mutations (Watanabe et al., 2010).

If ER is shown to be prognostically important in CKD, there are potential therapeutical applications: antiarrhythmic drugs such as isoproterenol and quinidine have been shown to reduce ER or even restore normal ECG patterns (Haissaguerre et al., 2009). Further, this electrocardiographic marker could contribute to risk stratification tools to guide ICD therapy in high risk patients.

### Study limitations

There are a number of limitations to the current study. The sample size was relatively small with a low incidence of mortality and progression to dialysis. This may have concealed any potential adverse effects of having ER. The retrospective design of the study did not permit the demonstration of any potential temporal relationships between CKD and ER or adverse clinical events. There was a difference between the ethnic origin of the patients with CKD and the RTD that were used as controls. This may have magnified the differences in prevalence of ER between the two groups with prior studies having shown an increased prevalence of ER in patients of Afro-Caribbean origin. Furthermore, a significant proportion of our cohort had a previous cardiac history, which has been associated with ER. Therefore, we may have overestimated the prevalence of ER in our population. However, on closer analysis of these patients, we found no significant association between previous cardiac history and the presence of ER. Finally owing to the study design, only a single ECG was available for analysis for each patient and it is unknown whether the presence of ER was a fixed or more dynamic electrocardiographic finding during the study period. Further studies are indicated to confirm our findings in a larger cohort of patients with CKD and also to assess for serial electrocardiographic changes.

## Conclusions

Electrocardiographic early repolarization is more common in patients with pre-dialysis CKD compared to healthy RTD. However, this increased prevalence of ER was not associated with increased 1-year mortality or entry onto dialysis programs. Further longitudinal studies are indicated to determine whether this increased prevalence of ER is associated with the increased rate of SCD observed in this population.

### Conflict of interest statement

The authors declare that the research was conducted in the absence of any commercial or financial relationships that could be construed as a potential conflict of interest.

## Abbreviations

CKD: chronic kidney disease
ECG: 12-lead electrocardiogram
ER: early repolarization
SCD: sudden cardiac death.
